# Neonicotinoid exposure in Tricolored Blackbirds (*Agelaius tricolor*)

**DOI:** 10.1007/s11356-022-23290-4

**Published:** 2022-09-28

**Authors:** Emily E. Graves, Robert J. Meese, Marcel Holyoak

**Affiliations:** Environmental Science & Policy Department, University of California, Davis, One Shields Avenue, Davis, CA 95616 USA

**Keywords:** Neonicotinoids, Insecticides, Pesticides, Birds, Non-target species, Liver, Conservation, Tricolored Blackbird, *Agelaius tricolor*

## Abstract

There is increasing awareness of the negative ecological and environmental effects of widespread use of pesticides on the landscape. Spillover or drift of pesticides from agricultural areas has been shown to impact species health, reproduction, and trophic dynamics through both direct and indirect mechanisms. Neonicotinoid insecticides are associated with observed declines of insectivorous and grassland birds, and these environmental pollutants are a significant conservation concern for many species that have experienced past or current population declines. Due to the high efficacy of these modern insecticides in depressing local insect populations, insectivorous birds can be negatively impacted by a pesticide-mediated reduction in food supply. Neonicotinoids may act synergistically with other stressors, such as habitat loss, to exacerbate threats to species or population viability. The Tricolored Blackbird is an insectivorous grassland bird of conservation concern in California, USA. Due to the high association of this species with agricultural habitats, we sought to quantify the amount of neonicotinoid residues in Tricolored Blackbird carcasses as a first step in assessing how this species may be impacted by pesticides. Out of 85 salvaged carcasses sampled (*N* = 24 adults, *N* = 3 fledglings, and *N* = 58 nestlings), only two contained detectable levels of target compounds. These were an adult and one nestling that contained clothianidin residue (40 ppb and 7 ppb, respectively); both of these birds were salvaged from breeding colonies associated with dairy farms in Kern County, California. We suggest that further work is needed to assess neonicotinoid exposure of Tricolored Blackbirds in dairy-associated breeding colonies.

## Introduction

### Ecological effects of neonicotinoids

Pesticides are widely used to meet the demands of the global food supply, although there are myriad examples of the detrimental effects of pesticides as an environmental pollutant on water quality, biodiversity, and even human health (Tang et al. 2021). The use of pesticides and other synthetic chemicals across the globe has increased rapidly over the last several decades, and much is known about the effects of pesticides on target pest species. However, research on these practices as a major contributor to global change has until recently been overlooked (Bernhardt et al. 2017). It is now clear that pesticide use can result in negative ecological effects such as declines in biodiversity and a reduction of biological pest control (Geiger et al. 2010; Hallmann et al. 2017; Møller et al. 2021). There is evidence that biodiversity declines due to habitat loss or conversion to agriculture are exacerbated by agricultural pesticide use (Gibbs et al. 2009; Tsiafouli et al. 2015).

A new class of pesticides, neonicotinoid insecticides, were developed in the 1980s and since then it has become the most widely used class of insecticides in the world (Goulson 2013). The neonicotinoid imidacloprid is one of the most commonly applied pesticides across the globe (Jeschke et al. 2011). Neonicotinoids are applied in both agricultural and home garden settings as seed coatings, foliar sprays, soil drenches, and granules (Hladik et al. 2018). They offer long-lasting protection against insect herbivory as a systemic pesticide, as the chemicals are integrated into tissues through entire plants during growth (Goulson 2013). Neonicotinoids are particularly toxic to insects by acting as a powerful nicotinic acetylcholine receptor blocker (Matsuda et al. 2001; Pisa et al. 2015; Tomizawa et al. 2000). On average, just 5% of neonicotinoids applied as seed coatings are actually taken up by the target plant, leaving 95% of the compound in the surrounding soil and water (Sur and Stork 2003). This runoff introduces neonicotinoids into the environment surrounding agricultural areas, where they can remain persistent for long periods of time under certain conditions (Bonmatin et al. 2015; Hladik et al. 2018). These compounds have demonstrated negative impacts on aquatic invertebrate biomass at levels below government regulatory compliance standards (Schepker et al. 2020), further supporting how these chemicals can have significant indirect ecological effects even at low concentrations. Early in 2022, the California Department of Pesticide Regulation filed an official notice of formal rulemaking to restrict the use of imidacloprid, thiamethoxam, clothianidin, and dinotefuran in California in an effort to protect pollinator health (DPR Regulation No. 22–001).

Neonicotinoid pesticides have been studied frequently for their negative effects on pollinating insects, specifically the European Honey Bee (*Apis mellifera*) which is a critical pollinator for many agricultural crops (Godfray et al. 2015; Henry et al. 2012; Woodcock et al. 2017). Because of the importance of pollination as an ecosystem service, there has been concern over the connection between neonicotinoid use and an overall decline of bee populations in recent years (Fairbrother et al. 2014; Henry et al. 2012). Pesticide pollution is generally considered to be a major driver in the ongoing declines of global insect populations (Sánchez-Bayo and Wyckhuys 2019). For example, populations of butterflies have also been shown to be impacted by spill-over of pesticides into non-agricultural habitats (Forister et al. 2016).

### Impacts of neonicotinoids on birds

The acute toxicity of neonicotinoids to birds is relatively low when compared to other classes of pesticides (such as organophosphates or carbamates) that have been largely replaced by neonicotinoids (Mineau and Palmer 2013). However, a growing body of literature suggests that neonicotinoids have negative indirect effects (e.g., food chain disruptions) on birds, in addition to potential direct acute toxicity (Goulson 2014; Hallmann et al. 2014). Recent analyses suggest that the use of neonicotinoid pesticides is associated with declines in bird populations at large continental/regional scales (Goulson 2014; Tallamy and Shriver 2021), with insectivorous birds impacted to the greatest extent through a pesticide-mediated reduction in food supply (Hallmann et al. 2014; Møller et al. 2021; Wilson et al. 1999). Thus, neonicotinoid pesticides appear to indirectly impact non-target species at exposure concentrations well below the amount that would induce acute toxic effects such as death in vertebrates (Goulson 2014).

One of the earliest studies to demonstrate the effects of pesticides on the population declines of a single bird species found that declines in Grey Partridges (*Perdix perdix*) was linked directly to declines in arthropod prey due to insecticide application (Potts 1986). Another early field study found that mean brood size and insect abundance were both significantly higher in unsprayed fields compared to sprayed fields (Rands 1985). Field experiments have identified relationships between invertebrate abundance and chick condition or survival in passerines as well (Boatman et al. 2004). In Yellowhammers (*Emberiza citronella*), brood reduction was more likely to occur when a greater proportion of surrounding foraging areas had been sprayed with insecticides (Boatman et al. 2004). There was also a negative relationship between insecticide use and Yellowhammer nestling body condition and a negative relationship between insecticide use and invertebrate prey abundance (Morris et al. 2005).

Notwithstanding their negative effects, neonicotinoid use in wild bird habitat is extremely widespread, and exposure is ubiquitous in agricultural habitats across many bird taxa. A recent study found that every collected sample of House Sparrow feathers contained at least one neonicotinoid compound, and samples from conventional farms had significantly higher concentrations than samples from organic farms (Humann-Guilleminot et al. 2019a). Another recent investigation found that 69% of Barn Owl (*Tyto alba*) nestling feathers and 57% of Barn Owl adult feathers contained at least one neonicotinoid compound (Humann-Guilleminot et al. 2021). This same study found no neonicotinoid residue in Alpine Swift (*Tachymarptis melba*) nestling feathers, but did find that 75% of food boluses and 20% adult plasma samples contained at least one neonicotinoid compound, indicating a diversity of possible exposure routes (Humann-Guilleminot et al. 2021). Further demonstrating the ubiquity of these chemicals in the environment, 100% of Mediterranean Gull (*Ichthyaetus melanocephalus*) and 89% of Sandwich Tern (*Thalasseus sandvicensis*) fledgling feather samples contained one neonicotinoid compound (Distefano et al. 2022).

Debilitation such as ataxia can be induced in birds given imidacloprid orally at an order of magnitude below the lethal dose (Callahan and Mineau 2008). These chemicals can cause disruption of endocrine and immune functions and induce changes in feeding behavior (Mitra et al. 2011). Acute neonicotinoid (imidacloprid) exposure in White-crowned Sparrow (*Zonotrichia leucophrys*) has been shown to induce decreased fat stores, lower body mass, and improper migratory orientation (Eng et al. 2017). Fertility may also be reduced at sublethal doses of neonicotinoids; House Sparrows (*Passer domesticus*) that were given a field-realistic dose of acetamiprid showed a significant reduction is sperm density (Humann-Guilleminot et al. 2019b). Overall, there is sufficient evidence from both laboratory and field studies to demonstrate that neurotoxic neonicotinoid pesticides can have detrimental direct and indirect effects on bird reproduction, foraging, and predator avoidance (Walker 2003).

### Declines of grassland birds

Changes in bird diversity can be an early warning of environmental problems (Arya et al. 2019) and massive declines in avian abundance over the last half century or more have been well established (Rosenberg et al. 2019). When controlled for the effects of agricultural intensification and land-use change, declines of grassland birds in particular have been linked to the widespread use of pesticides. A review of agricultural drivers of farmland-associated bird species in North America found that 42% of studies found a negative impact of pesticides, while 27% of studies found a negative impact of habitat loss (Stanton et al. 2018). Neonicotinoid pesticide use in the USA was associated with a 4% annual decline of grassland birds and a 3% annual decline of insectivorous birds (Li et al. 2020). Overall, grassland birds have declined by 53% since the 1970s, faster than any other group (Rosenberg et al. 2019). Pesticides are estimated to affect 87% of bird species that are threatened globally, with a disproportionate impact on grassland birds (Arya et al. 2019).

For endangered species across taxa, the use of population models in assessing pesticide risk for listed species has been extremely limited (Forbes et al. 2016). Pesticide exposure, even if low, can cause additional pressure to species that are already declining. Attempts to include pesticide exposure into models of avian survival and reproduction have been limited by the availability of direct controlled toxicological studies (Bennett et al. 2007; Etterson and Bennett 2013). A lack of toxicological data for many wild species is, in part, responsible for the lack of information of how pesticides may impact species of conservation concern across temporal and geographic scales (Forbes et al. 2016). Thus, establishing exposure (as the presence of pesticides in tissue) is a crucial first step in evaluating the potential effects of neonicotinoids on species of conservation concern. In this study, we seek to establish this baseline for pesticide exposure in a grassland bird of conservation concern in California, USA.

### Tricolored Blackbirds as a study species

The Tricolored Blackbird is a highly colonial marsh-nesting songbird that is nearly endemic to California (Neff 1937) and has experienced drastic population declines in recent years (Graves et al. 2013; Meese 2013; Robinson et al. 2021). The Tricolored Blackbird is listed as Threatened under the California Endangered Species Act and is designated as Endangered by the IUCN Red List. The species is the most colonial land bird in North America since the extinction of the Passenger Pigeon (*Ectopistes migratorius*; Bent 1958), and breeding in high-density large colonies makes the species especially vulnerable to dramatic nesting failures (Cook and Toft 2005). Tricolored Blackbirds also exhibit semi-nomadic behavior and itinerant breeding (Hamilton 1998; Orians 1961). Historically, over 90% of known individuals nested in wetlands and foraged primarily in grasslands (DeHaven et al. 1975; Neff 1937; Orians 1961). Wetland habitats have experienced losses of over 90% in California’s Central Valley (Frayer et al. 1989). However, potential positive signs are shown by the species nesting in upland non-native vegetation and agricultural habitats with increasing frequency and density over the last several decades (Meese 2017). Neonicotinoid use in California has been linked to population declines in Tricolored Blackbirds and Purple Martins (*Progne subis*; Forister et al. 2016). Due to the high association of Tricolored Blackbirds with agricultural areas, especially silage fields for dairy cattle, we sought to investigate pesticide residues in Tricolored Blackbirds to establish pesticide exposure risk across different land-use types. We expect that birds in agricultural areas will have higher measured pesticide residue levels than birds breeding in non-agricultural areas.

## Methods

### Study sites and sample collection

Tricolored Blackbird carcasses were opportunistically salvaged from breeding colony locations during banding and monitoring efforts from April through the beginning of July during 2017–2020. Adult (*N* = 24) and fledgling (*N* = 3) carcasses were found as a result of vehicle collisions or birds striking the windows of buildings. Nestling carcasses (*N* = 58) were obtained as a result of brood reduction behavior that is commonly observed in this species, where parents will deposit live or dead nestlings along the perimeters of their breeding colonies. No birds were killed as a part of this study. Nestlings are obligate insectivores and dependent on local insect populations at this stage of life, and because they are still in the nest we know that any insecticide exposure came from the local area. By comparison, adults are partially granivorous and pesticide exposure may have occurred elsewhere during earlier time periods.

**Fig. 1:**
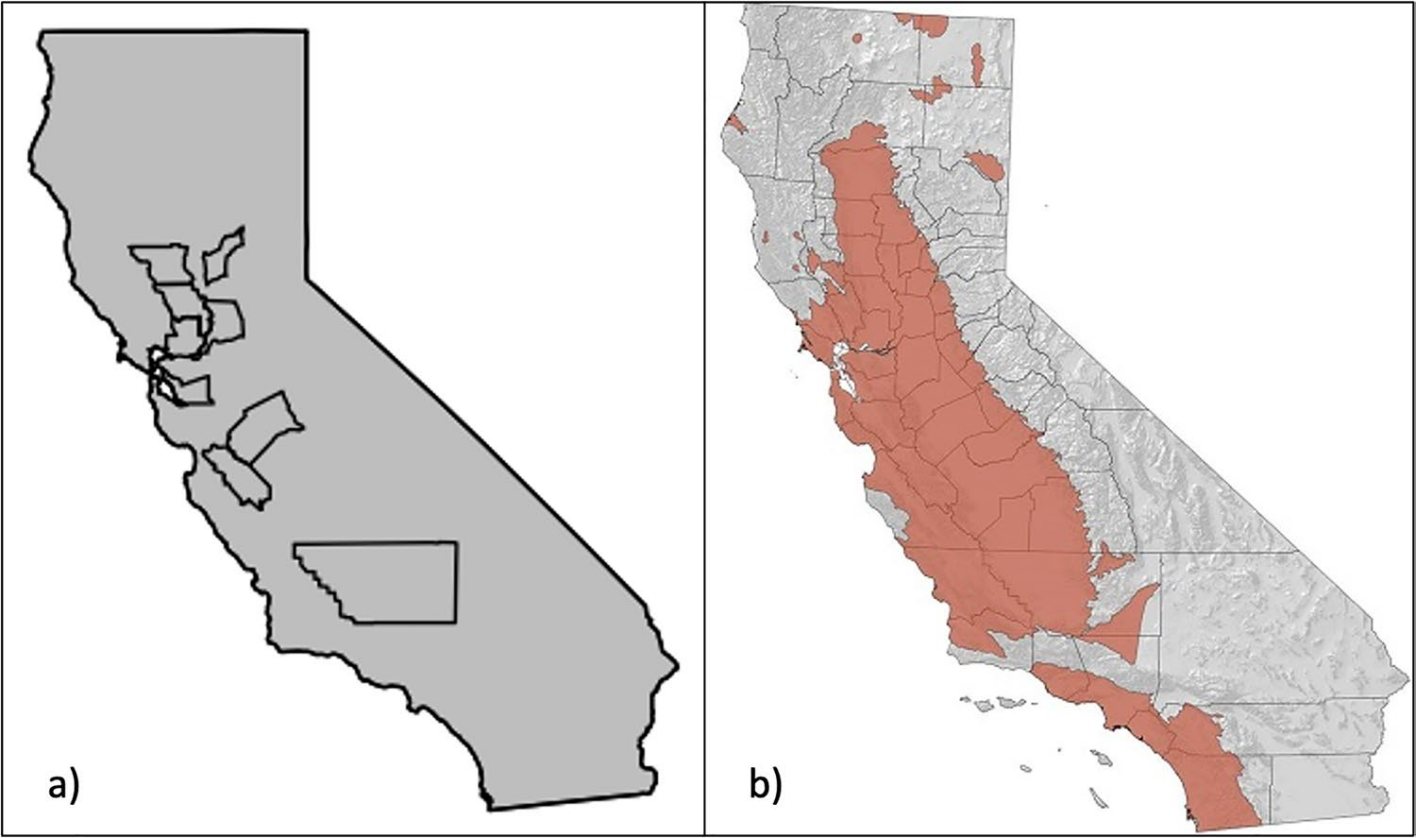
**a** Map of California showing the counties where adult and nestling Tricolored Blackbird carcasses were salvaged; **b** range map of the Tricolored Blackbird in California (shown in red; obtained from the California Department of Wildlife; https://wildlife.ca.gov/).

We obtained carcasses from scattered counties across the core of the species’ range in California: Alameda (*N* = 1), Colusa (*N* = 9), Kern (*N* = 2), Merced (*N* = 5), Sacramento (*N* = 15), San Benito (*N* = 4), Solano (*N* = 1), Yolo (*N* = 16), and Yuba (*N* = 32). Figure 1 shows the counties sampled relative to the species range in California. Samples were collected across four field seasons: 2017 (*N* = 19), 2018 (*N* = 6), 2019 (*N* = 49), and 2020 (*N* = 11). Sample collection in 2020 was limited due to travel restrictions caused by the Covid-19 pandemic. Upon collection in the field, samples were immediately put into an ice chest for transportation, and then transferred to a − 80 °C freezer for storage until the pesticide assays were performed. Nestling and adult carcasses were salvaged in as fresh a state as possible (i.e., no signs of decomposition), but the exact time period since death is unknown because of the opportunistic nature of this study. Liver tissues (whole liver) were extracted from the adult carcasses prior to pesticide analysis and the nestling carcasses remained whole.

### Pesticide analysis

A liquid chromatography-mass spectrometry (LC-HRMS) assay has been developed and validated to detect neonicotinoid pesticides and other compounds in small-body avian tissue samples using homogenized carcasses of 1–2 day-old chicken carcasses (Filigenzi et al. 2019). This method has been successfully used to document insecticide exposure in free-ranging hummingbirds in California (Graves et al. 2019). The method allows for the analysis of pesticide residues in small-bodied species where traditional methods of sampling (i.e., liver tissue or blood sampling) are not possible. Given the small size of Tricolored Blackbird nestlings, the present pesticide analyses were done according to the whole-carcass methods described in Filigenzi et al. (2019) and Graves et al. (2019). The same LC-HRMS assay was performed on the adult liver tissue samples. Target compounds for these assays were dinotefuran, nitenpyram, thiamethoxam, clothianidin, imidacloprid, acetamiprid, thiacloprid, and sulfoxaflor. Analyses were conducted by staff at the California Animal Health and Food Safety Laboratory at the School of Veterinary Medicine, University of California, Davis, CA 95616.

## Results

Out of the 85 birds sampled, only 2 carcasses contained residues of any target compound above the detection limit. Clothianidin was the only target compound detected. One adult male liver showed 40 ppb of clothianidin and one 7-day-old nestling carcass showed 7.1 ppb of clothianidin (limit of quantification 1.0 ppb).

Both birds with detectable levels of clothianidin were salvaged from areas adjacent to breeding colonies located in silage fields associated with dairy farms in Kern County, California. These two carcasses were also the only 2 samples obtained from Kern County. With only 7 total samples able to be salvaged from dairy/silage habitat in Kern and Merced Counties, we observed 2 carcasses with clothianidin residue (28.6%). The other 78 samples from non-silage breeding colonies contained no detectable levels of any target compound (0%). With only two samples above the detection limit, there would be very low statistical power in any statistical comparison of frequency of detection in different counties or regions, and therefore we did not attempt a statistical analysis of the findings.

## Discussion

Our low detection rates (2 out of 85 birds sampled) of neonicotinoids is surprising considering Tricolored Blackbirds’ common association with agricultural habitats during the breeding season. However, the two carcasses with detectable levels of clothianidin were both salvaged from breeding colonies in silage fields associated with dairies; no bird samples from other land-use types showed any pesticide residue. Clothianidin was the only target compound detected in any of our samples. The EPA characterizes clothianidin as “moderately toxic to birds on an acute oral exposure basis” and “practically nontoxic on a subacute dietary exposure basis” (EPA 2020). Clothianidin has however been shown to cause eggshell thinning (EPA 2020). Thiamethoxam is known to metabolize into clothianidin when given orally to birds (Pan et al. 2022), so our detection of clothianidin may be a metabolite following initial environmental exposure to thiamethoxam (rather than environmental exposure to clothianidin). Thiamethoxam has negative impacts on commercial laying hen productivity, with sub-lethal doses causing eggshell thinning, anemia, reduced food consumption, and damage to the liver and kidneys (Gul et al. 2020). Sub-lethal doses of thiamethoxam also have toxic effects on hematological and biochemical parameters in broiler chicks (Gul et al. 2018). More research is needed to show if exposure to clothianidin or thiamethoxam has measurable impact on Tricolored Blackbird reproduction or physiology for individuals nesting in silage fields.

Birds may be coming into contact with our target compounds at a frequency that was not captured by our salvage sampling method. Environmental exposure may be higher at other times of the year outside the Tricolored Blackbird breeding season. Surveillance of clothianidin exposure in European gamebirds demonstrated a significant seasonal difference, with only 6% of birds showing detectable residues before sowing with treated seeds compared to 89% of samples after sowing (Lennon et al. 2020). Laboratory studies have shown that birds rapidly eliminate neonicotinoids from the body. Japanese Quail (*Coturnix japonica*) that have been orally dosed with imidacloprid rapidly absorb the compound into blood, brain, liver, and kidney tissues (within 1 h) but eliminate the compound to below the detection threshold within 24 h (Bean et al. 2019). Similar rapid rates of clearance in Japanese Quail have been shown with thiamethoxam and clothianidin (Pan et al. 2022). Neonicotinoids are known to persist in the environment for long periods of time (Bonmatin et al. 2015), so it is unlikely that our target compounds broke down in the salvaged carcasses prior to storage at − 80 °C.

There is some evidence to suggest that birds preferentially avoid seeds treated with neonicotinoids (Lopez-Antia et al. 2014). A study of eight Ring-necked Pheasants (*Phasianus colchicus*) found that given the choice of untreated, dyed, and dyed/treated seed corn (treated with Poncho® 1250 clothianidin), birds selected (*p* < 0.0001) untreated seeds over dyed and treated seeds (Sundall 2020). If treated seeds are the route of dietary exposure for adult Tricolored Blackbirds and birds are actively avoiding this food source, this may help explain why we are seeing low or no pesticide residue in adult samples. However, further research is needed to identify the method of exposure to clothianidin (or thiamethoxam) in this species, particularly for nestlings.

In summary, we report the first application of a direct pesticide residue analysis to quantify the field exposure of Tricolored Blackbirds to neonicotinoid pesticides during the breeding season. Of 85 opportunistically collected birds, two (an adult and a nestling) showed the presence of clothianidin only. Both came from breeding colonies associated with dairies in Kern County, and were two of only seven bird carcasses salvaged from dairy-associated colonies. The other 78 carcasses salvaged from other (non-silage) breeding habitat locations throughout the species range contained no detectable levels of any target neonicotinoid insecticides. As of the last statewide population survey in 2017, 34.4% of breeding Tricolored Blackbirds nest in Kern County, which is a higher proportion than in any other county (Meese 2017). Survey results also show that 33.1% of Tricolored Blackbirds nest in substrates associated with silage (Meese 2017). Additional targeted sampling efforts are needed to further explore the potential impacts of insecticides on Tricolored Blackbird breeding in this geographic area and also in this breeding substrate type. Pesticide exposure occurring outside of the breeding season and/or in non-breeding birds would not have been captured in our study, so further investigation is needed to identify additional possible routes of exposure across the Tricolored Blackbird annual cycle. Additionally, our detection rates in adults may have been different if blood or feather samples were taken from adults rather than liver samples. Blood and feather samples may show a different exposure route than that would be detected in liver tissue. This study only targeted neonicotinoid insecticides (and sulfoxaflor, a sulfoximine systemic insecticide), so further evaluation of exposure to other classes of pesticides is also necessary. Additional research is needed to understand if and how insecticide application affects the insect prey base of Tricolored Blackbirds, as these indirect effects are known to play a large role in the ongoing declines of grassland and insectivorous birds.

## Data Availability

All data used is presented and available within this manuscript.

## References

[CR1] Arya AK, Singh A, Bhatt D (2019) Pesticide applications in agriculture and their effects on birds: an overview, in: Contaminants in Agriculture and Environment: Health Risks and Remediation. Agro Environ Media – Agric Environ Sci Acad, Haridwar, India, pp 129–137. 10.26832/AESA-2019-CAE-0163-010

[CR2] Bean TG, Gross MS, Karouna-Renier NK, Henry PFP, Schultz SL, Hladik ML, Kuivila KM, Rattner BA (2019). Toxicokinetics of imidacloprid-coated wheat seeds in Japanese quail ( *Coturnix japonica* ) and an evaluation of hazard. Environ Sci Technol.

[CR3] Bennett RS, Etterson MA, Bennett RS, Etterson MA (2007) Incorporating results of avian toxicity tests into a model of annual reproductive success 3, 498–507. 10.1897/IEAM 10.1897/ieam_2007-029.1 18046799

[CR4] Bent AC (1958) Life histories of North American blackbirds, orioles, tanagers, and allies. Bull US NatlMus 1–549. 10.5479/si.03629236.211.1

[CR5] Bernhardt ES, Rosi EJ, Gessner MO (2017). Synthetic chemicals as agents of global change. Front Ecol Environ.

[CR6] Boatman ND, Brickle NW, Hart, JD, Milsom TP, Morris AJ, Murray AWA, Robertson PA (2004) Evidence for the indirect effects of pesticides on farmland birds. Ibis 146:131–143

[CR7] Bonmatin J-M, Giorio C, Girolami V, Goulson D, Kreutzweiser DP, Krupke C, Liess M, Long E, Marzaro M, Mitchell EAD, Noome DA, Simon-Delso N, Tapparo A (2015). Environmental fate and exposure; neonicotinoids and fipronil. Environ Sci Pollut Res.

[CR8] Callahan J, Mineau P (2008). An evaluation of clinical sign data from avian acute oral toxicity studies. Appendix 11; Scientific opinion of the Panel on Plant Protection Products and their Residues on Risk Assessment for Birds and Mammals. EFSA J.

[CR9] Cook LF, Toft CA (2005). Dynamics of extinction: population decline in the colonially nesting Tricolored Blackbird Agelaius tricolor. Bird Conserv Int.

[CR10] DeHaven RW, Crase FT, Woronecki PP (1975). Movements of tricolored blackbirds banded in the Central Valley of California, 1965–1972. Bird-Band.

[CR11] Distefano GG, Zangrando R, Basso M, Panzarin L, Gambaro A, Volpi Ghirardini A, Picone M (2022). The ubiquity of neonicotinoid contamination: residues in seabirds with different trophic habits. Environ Res.

[CR12] Eng ML, Stutchbury BJM, Morrissey CA, Mineau P, Whiteside M, Jeschke P, Nauen R, Schindler M, Elbert A, Tomizawa M, Casida J, Čolović M, Krstić D, Lazarević-Pašti T, Bondžić A, Vasić V, Hallmann C, Foppen R, Turnhout C, Kroon H, Jongejans E, Lopez-Antia A, Ortiz-Santaliestra M, Mougeot F, Mateo R, Gibbons D, Morrissey C, Mineau P, Lopez-Antia A, Ortiz-Santaliestra M, Mougeot Fo, Mateo R, Smith A, McWilliams S, Alerstam T, Chernetsov N, Mouritsen H, Heyers D, Güntürkün O, Grue C, Gibert P, Seeley M, Narvaez C, Rios J, Piriz G, Sanchez-Hernandezc J, Sabat P, Newton I, Prosser P, Nattrass C, Prosser C, Vyas N, Balcomb R, Brasel J, Collier A, Pritsos C, Moye J, Pritsos C, Vyas N, Hill E, Sauer J, Kuenzel W, Brennan L, Kuvlesky W, Geiger F, Lopez-Antia A, Ortiz-Santaliestra M, Mateo R, Avery M, Decker D, Fischer D, Stafford T, Gómez C, Kokko H, Smith R, Moore F, Rattenborg N, Singh J, Rastogi A, Rani S, Kumar V, Ramenofsky M, Agatsuma R, Ramfar T, Lopez-Antia A, Feliu J, Camarero P, Ortiz-Santaliestra M, Mateo R, Solomon K, Best L, Gionfriddo J, Tscharntke T, Klein A, Kruess A, Steffan-Dewenter I, Thies C, Agatsuma R, Ramenofsky M, Muheim R, Henshaw I, Sjöberg S, Deutschlander M (2017). Imidacloprid and chlorpyrifos insecticides impair migratory ability in a seed-eating songbird. Sci Rep.

[CR13] Etterson MA, Bennett RS (2013) Quantifying the effects of pesticide exposure on annual reproductive success of birds 9, 590–599. 10.1002/ieam.1450 10.1002/ieam.1450 23728843

[CR14] Fairbrother A, Purdy J, Anderson T, Fell R (2014). Risks of neonicotinoid insecticides to honeybees. Environ Toxicol Chem.

[CR15] Filigenzi MS, Graves EE, Tell LA, Jelks KA, Poppenga RH (2019). Quantitation of neonicotinoid insecticides, plus qualitative screening for other xenobiotics, in small-mass avian tissue samples using UHPLC high-resolution mass spectrometry. J Vet Diagn Invest.

[CR16] Forbes VE, Galic N, Schmolke A, Vavra J, Pastorok R, Thorbek P (2016). Assessing the risks of pesticides to threatened and endangered species using population modeling: a critical review and recommendations for future work: population modeling of pesticide risks to listed species. Environ Toxicol Chem.

[CR17] Forister ML, Cousens B, Harrison JG, Anderson K, Thorne JH, Waetjen D, Nice CC, De Parsia M, Hladik ML, Meese R, van Vliet H, Shapiro AM (2016). Increasing neonicotinoid use and the declining butterfly fauna of lowland California. Biol Lett.

[CR18] Frayer WE, Peters DD, Pywell WR (1989) Wetlands of the California Central Valley: status and trends 1939 to mid-1980’s. U.S. Fish and Wildlife Service, Portland, Oregon

[CR19] Geiger F, Bengtsson J, Berendse F, Weisser WW, Emmerson M, Morales MB, Ceryngier P, Liira J, Tscharntke T, Winqvist C, Eggers S, Bommarco R, Pärt T, Bretagnolle V, Plantegenest M, Clement LW, Dennis C, Palmer C, Oñate JJ, Guerrero I, Hawro V, Aavik T, Thies C, Flohre A, Hänke S, Fischer C, Goedhart PW, Inchausti P (2010). Persistent negative effects of pesticides on biodiversity and biological control potential on European farmland. Basic Appl Ecol.

[CR20] Gibbs KE, Mackey RL, Currie DJ (2009) Human land use , agriculture , pesticides and losses of imperiled species 242–253 10.1111/j.1472-4642.2008.00543.x

[CR21] Godfray HCJ, Blacquière T, Field LM, Hails RS, Potts SG, Raine NE, Vanbergen AJ, McLean AR (2015). A restatement of recent advances in the natural science evidence base concerning neonicotinoid insecticides and insect pollinators. Proc r Soc B Biol Sci.

[CR22] Goulson D (2013). REVIEW: an overview of the environmental risks posed by neonicotinoid insecticides. J Appl Ecol.

[CR23] Goulson D (2014). Pesticides linked to bird declines. Nature.

[CR24] Graves EE, Holyoak M, Rodd Kelsey T, Meese RJ (2013). Understanding the contribution of habitats and regional variation to long-term population trends in tricolored blackbirds. Ecol Evol.

[CR25] Graves EE, Jelks KA, Foley JE, Filigenzi MS, Poppenga RH, Ernest HB, Melnicoe R, Tell LA (2019). Analysis of insecticide exposure in California hummingbirds using liquid chromatography-mass spectrometry. Environ Sci Pollut Res.

[CR26] Gul ST, Khan A, Ahmad M, Anwar MF, Khatoon A, Saleemi MK, Akram MN (2018). Effect of sub-lethal doses of thiamethoxam (a neonicotinoid) on hemato-biochemical parameters in broiler chicks. Toxin Rev.

[CR27] Gul ST, Ahamd I, Saleemi MK, Ahmad M, Ahmad L, Khan A (2020). Toxico-pathological effects of thiamethoxam on hemato-biochemical and productive performance of commercial laying hens. Pak Vet J.

[CR28] Hallmann CA, Foppen RPB, van Turnhout CAM, de Kroon H, Jongejans E (2014). Declines in insectivorous birds are associated with high neonicotinoid concentrations. Nature.

[CR29] Hallmann CA, Sorg M, Jongejans E, Siepel H, Hofland N, Schwan H, Stenmans W, Müller A, Sumser H, Hörren T, Goulson D, de Kroon H (2017). More than 75 percent decline over 27 years in total flying insect biomass in protected areas. PLoS One.

[CR30] Hamilton WJ (1998). Tricolored blackbird itinerant breeding in California. The Condor.

[CR31] Henry M, Béguin M, Requier F, Rollin O, Odoux J, Aupinel P, Aptel J, Tchamitchian S, Decourtye A (2012). A common pesticide decreases foraging success and survival in Honey Bees. Science.

[CR32] Hladik ML, Main AR, Goulson D (2018). Environmental risks and challenges associated with neonicotinoid insecticides. Environ Sci Technol.

[CR33] Humann-Guilleminot S, Clément S, Desprat J, Binkowski ŁJ, Glauser G, Helfenstein F (2019). A large-scale survey of house sparrows feathers reveals ubiquitous presence of neonicotinoids in farmlands. Sci Total Environ.

[CR34] Humann-Guilleminot S, Tassin de Montaigu C, Sire J, Grünig S, Gning O, Glauser G, Vallat A, Helfenstein F (2019). A sublethal dose of the neonicotinoid insecticide acetamiprid reduces sperm density in a songbird. Environ Res.

[CR35] Humann-Guilleminot S, Laurent S, Bize P, Roulin A, Glauser G, Helfenstein F (2021). Contamination by neonicotinoid insecticides in barn owls (Tyto alba) and Alpine swifts (Tachymarptis melba). Sci Total Environ.

[CR36] Jeschke P, Nauen R, Schindler M, Elbert A (2011). Overview of the status and global strategy for neonicotinoids. J Agric Food Chem.

[CR37] Lennon RJ, Shore RF, Pereira MG, Peach WJ, Dunn JC, Arnold KE, Brown CD (2020). High prevalence of the neonicotinoid clothianidin in liver and plasma samples collected from gamebirds during autumn sowing. Sci Total Environ.

[CR38] Li Y, Miao R, Khanna M (2020). Neonicotinoids and decline in bird biodiversity in the United States. Nat Sustain.

[CR39] Lopez-Antia A, Ortiz-Santaliestra ME, Mateo R (2014). Experimental approaches to test pesticide-treated seed avoidance by birds under a simulated diversification of food sources. Sci Total Environ.

[CR40] Matsuda K, Buckingham SD, Kleier D, Rauh JJ, Grauso M, Sattelle DB (2001). Neonicotinoids: insecticides acting on insect nicotinic acetylcholine receptors. Trends Pharmacol Sci.

[CR41] Meese RJ (2013). Chronic low reproductive success of the colonial tricolored blackbird from 2006 to 2011. West Birds.

[CR42] Meese RJ (2017) Results of the 2017 tricolored blackbird statewide survey. California Department of Fish and Wildlife, Wildlife Branch, Nongame Wildlife Program

[CR43] Mineau P, Palmer C (2013) The impact of the nation’s most widely used insecticides on birds. American Bird Conservancy

[CR44] Mitra A, Chatterjee C, Mandal FB (2011) Synthetic chemical pesticides and their effects on birds. Res J Environ Toxicol 5(2):81–96

[CR45] Møller AP, Czeszczewik D, Flensted-Jensen E, Erritzøe J, Krams I, Laursen K, Liang W, Walankiewicz W (2021). Abundance of insects and aerial insectivorous birds in relation to pesticide and fertilizer use. Avian Res.

[CR46] Morris AJ, Wilson JD, Whittingham MJ, Bradbury RB (2005). Indirect effects of pesticides on breeding yellowhammer (Emberiza citrinella). Agric Ecosyst Environ.

[CR47] Neff JA (1937). Nesting distribution of the tri-colored red-wing. Condor.

[CR48] Orians GH (1961). The Ecology of Blackbird (Agelaius) Social Systems. Ecol Monogr.

[CR49] Pan Y, Chang J, Xu P, Xie Y, Yang L, Hao W, Li J, Wan B (2022). Twenty-four hours of thiamethoxam: in vivo and molecular dynamics simulation study on the toxicokinetic and underlying mechanisms in quails (Coturnix japonica). J Hazard Mater.

[CR50] Pisa LW, Amaral-Rogers V, Belzunces LP, Bonmatin JM, Downs CA, Goulson D, Kreutzweiser DP, Krupke C, Liess M, McField M, Morrissey CA, Noome DA, Settele J, Simon-Delso N, Stark JD, Van der Sluijs JP, Van Dyck H, Wiemers M (2015). Effects of neonicotinoids and fipronil on non-target invertebrates. Environ Sci Pollut Res.

[CR51] Potts GR (1986) The partridge: Pesticides, predation and conservation. Collins, London

[CR52] Rands MRW (1985). Pesticide use on cereals and the survival of grey partridge chicks: a field experiment. J Appl Ecol.

[CR53] Robinson OJ, Ruiz‐Gutierrez V, Meese RJ, Graves EE, Holyoak M, Wilson CR, Wyckoff AC, Merriell BD, Snyder C, Cooch EG (2021) Multi‐scale demographic analysis reveals range contraction via pseudo‐source and sink population structure. Ecosphere 12(5):1–6. 10.1002/ecs2.3521

[CR54] Rosenberg KV, Dokter AM, Blancher PJ, Sauer JR, Smith AC, Smith PA, Stanton JC, Panjabi A, Helft L, Parr M, Marra PP (2019). Decline of the North American avifauna. Science.

[CR55] Sánchez-Bayo F, Wyckhuys KAG (2019). Worldwide decline of the entomofauna: a review of its drivers. Biol Conserv.

[CR56] Schepker TJ, Webb EB, Tillitt D, LaGrange T (2020). Neonicotinoid insecticide concentrations in agricultural wetlands and associations with aquatic invertebrate communities. Agric Ecosyst Environ.

[CR57] Stanton RL, Morrissey CA, Clark RG (2018). Analysis of trends and agricultural drivers of farmland bird declines in North America: a review. Agric Ecosyst Environ.

[CR58] Sundall M (2020) The effect of the neonicotinoid clothianidin on ring-necked pheasant survival and reproduction. Master’s thesis, South Dakota State University

[CR59] Sur R, Stork A (2003). Uptake, translocation and metabolism of imidacloprid in plants. Bull Insectology.

[CR60] Tallamy DW, Shriver WG (2021). Are declines in insects and insectivorous birds related?. Ornithol Appl.

[CR61] Tang FHM, Lenzen M, McBratney A, Maggi F (2021). Risk of pesticide pollution at the global scale. Nat Geosci.

[CR62] Tomizawa M, Lee DL, Casida JE (2000). Neonicotinoid insecticides: molecular features conferring selectivity for insect versus mammalian nicotinic receptors. J Agric Food Chem.

[CR63] Tsiafouli MA, Thébault E, Sgardelis SP, de Ruiter PC, van der Putten WH, Birkhofer K, Hemerik L, de Vries FT, Bardgett RD, Brady MV, Bjornlund L, Jørgensen HB, Christensen S, Hertefeldt TD, Hotes S, Gera Hol WH, Frouz J, Liiri M, Mortimer SR, Setälä H, Tzanopoulos J, Uteseny K, Pižl V, Stary J, Wolters V, Hedlund K (2015). Intensive agriculture reduces soil biodiversity across Europe. Glob Change Biol.

[CR64] Walker CH (2003). Neurotoxic pesticides and behavioural effects upon birds. Ecotoxicology.

[CR65] Wilson JD, Morris AJ, Arroyo BE, Clark SC, Bradbury RB (1999). A review of the abundance and diversity of invertebrate and plant foods of granivorous birds in northern Europe in relation to agricultural change. Agric Ecosyst Environ.

[CR66] Woodcock BA, Bullock JM, Shore RF, Heard MS, Pereira MG, Redhead J, Ridding L, Dean H, Sleep D, Henrys P, Peyton J, Hulmes S, Hulmes L, Sárospataki M, Saure C, Edwards M, Genersch E, Knäbe S, Pywell RF (2017). Country-specific effects of neonicotinoid pesticides on honey bees and wild bees. Science.

